# Effectiveness of Non-Pharmacological Interventions for Irritable Bowel Syndrome: A Systematic Review

**DOI:** 10.1155/2021/4404185

**Published:** 2021-11-08

**Authors:** Florent Amsallem, Stéphane Sanchez, Xavier Armoiry, François Mion

**Affiliations:** ^1^INSERM, U1032 LABTAU, Université Claude Bernard Lyon 1, 69000 Lyon, France; ^2^Unité de Recherche Clinique et Recherche en Soins, CH de Troyes, 10000 Troyes, France; ^3^Faculté de Pharmacie (ISPB), UMR CNRS 5510 MATEIS, Université de Lyon, 69000 Lyon, France; ^4^Hôpital Edouard Herriot, Pharmacy Department, 69003 Lyon, France; ^5^Division of Health Sciences, University of Warwick, Coventry, UK; ^6^Université Lyon 1, Physiology Department, 69003 Lyon, France; ^7^Hospices Civils de Lyon, Digestive Physiology Department, Hôpital Edouard Herriot, 69003 Lyon, France

## Abstract

**Introduction:**

Given the complexity of the therapeutic management of irritable bowel syndrome (IBS), alternative non-pharmacological therapies are frequently offered to patients. The aim of this study was to conduct a systematic review in order to establish the current evidence base for non-pharmacological interventions (body-directed and mind-body therapies) in the management of IBS.

**Materials and Methods:**

The literature was searched in several electronic databases (PubMed (including Medline), Web of Science (Clarivate Analytics), Scopus (Elsevier), ScienceDirect (Elsevier), Cochrane Library (Wiley), and Wiley Online Library (Wiley)) for randomized controlled trials (RCTs) published in the English language from 1990 to 2020. Effectiveness outcomes were examined through the change in overall IBS symptoms or abdominal pain up to 12 months after treatment.

**Results:**

11 studies (parallel-group RCTs) were identified that enrolled 1590 participants in total. Body-directed therapies (acupuncture and osteopathic medicine) showed a beneficial effect compared with standard medical treatment for overall IBS symptoms at 6 months follow-up, while no study found any difference between body-directed and sham therapies for abdominal pain or overall IBS symptoms. It was not possible to conclude whether hypnotherapy was superior to standard medical treatment or supportive therapy for overall IBS symptoms or abdominal pain due to discordant results.

**Conclusions:**

Although body-directed therapies such as acupuncture and osteopathic medicine may be beneficial for overall IBS symptoms, higher-quality RCTs are needed to establish the clinical benefit of non-pharmacological interventions for IBS. An important challenge will be the definition of the optimal control groups to be used in non-pharmacological trials.

## 1. Introduction

Irritable bowel syndrome (IBS) is a chronic functional disorder of the gut characterized by abdominal pain associated with changes in the consistency and/or frequency of bowel movements. With a global prevalence of 5 to 10% according to the Rome IV criteria [1], a high impact on the rate of work absenteeism, high healthcare costs, and a sharp reduction in quality of life due to chronicity of symptoms [2–4], IBS has led to increased use of conventional medical care, at an estimated cost of hundreds of billions of dollars/year [5]. IBS symptoms are also associated with mild-to-severe anxiety and depression in two-thirds of patients [6]. Given the significant demand for care from IBS patients, physicians generally prescribe symptomatic drug therapies, but these have only modest effectiveness [7]. Indeed, the multifactorial nature of IBS physiopathology, the wide variation in symptoms in terms of severity and progression, and the fairly significant placebo effect may constrain the development of more effective drugs [7–9].

This complex clinical context and the low effectiveness of available medications prompt many patients to use complementary and alternative medicines, such as mind-body therapies (hypnotherapy, mindfulness/meditation, yoga, etc.), body-directed therapies (osteopathic medicine, chiropractic care, acupuncture, reflexology, etc.), dietary supplements, and energy therapies. Phytotherapy may have potential beneficial effects, as recently suggested for curcumin by a meta-analysis [10]. Spanier et al. reported the use of alternative treatments by 11–43% of patients, who reported satisfaction with these approaches [11]. It is estimated that nearly 50% of IBS patients use complementary medicines [12, 13], with 59% who use acupuncture [14]. A study conducted in the United States revealed that among 1409 subjects evaluated, 50.9% used these treatments in IBS [15].

Regarding body-directed therapies, the advent of safety data on acupuncture [16–18] and the limited availability of other safe and effective treatments for IBS raise the question of the effectiveness of acupuncture in the treatment of IBS. Concurrently, a therapeutic benefit of osteopathic medicine in 200 patients meeting the Rome III criteria has been reported [19]. There has also been interest in mind-body therapies to complement or replace medicine, including cognitive behavioral therapy and hypnotherapy [20]. Hypnotherapy became popular in the 1980s, when an RCT demonstrated some therapeutic effectiveness [21]. In recent years, hypnotherapy has shown a beneficial impact on IBS symptoms [22–34] and according to Billings et al. [35], mind-body therapies, dietary supplements, and herbal medicine have been shown to be beneficial for abdominal pain and overall IBS symptoms in a setting where evidence of effectiveness is weak.

There is a growing body of literature on the use of complementary and alternative medicine, including NPIs. However, no systematic review has specifically examined the evidence for the use of non-pharmacological, body-directed, and mind-body interventions (together termed NPIs). A systematic review of these NPIs in the management of IBS was conducted. The specific objective was to measure their effectiveness and to discuss their role in the treatment of IBS.

## 2. Materials and Methods

This review was performed in compliance with the Preferred Reporting Items for Systematic Reviews and Meta-Analyses (PRISMA) updated guidelines [36].

To be included, studies had to be RCTs. The population of interest was adults and children with functional bowel disorders (IBS, functional constipation, and functional diarrhea). In the included studies, all the patients had a formal clinical diagnosis of IBS. The interventions of interest, namely, NPIs, were body-directed therapies (osteopathic medicine, chiropractic care, traditional Chinese acupuncture, auriculotherapy, and reflexology) and mind-body therapies (hypnotherapy). Hypnotherapy is based on hypnosis techniques, making it possible to focus attention and generate strong suggestibility in the subject. Acupuncture consists in the application of very fine needles under the skin to restore vital energy. Auriculotherapy considers the existence of a correspondence between the external auricle (outer portion of the ear) and the organs of the body. It consists in applying sterile needles and electrical stimulation to the auricle. Osteopathic medicine consists in performing techniques on different anatomical areas to restore a state of normotony between structure and function. Chiropractic care is based on the manipulative treatment of lesioned joints, especially those of the spine, which are involved in the development of disorders that affect organs, muscles, and other tissues. Reflexology is a massage technique aimed at stimulating reflex zones to act remotely on painful organs.

The comparators selected were sham procedures and/or standard treatments. The main exclusion criteria were nonrandomized studies, patients without a formal diagnosis of IBS, and waiting-list controls (comparators). Effectiveness outcomes were self-reported global gastrointestinal score (continuous variable), self-reported adequate symptom relief (dichotomous variable: responder vs. nonresponder), and abdominal pain up to 12 months of follow-up.

Search strategies were developed with the assistance of a librarian from the Central Documentation Department of the Hospices Civils de Lyon with expertise in systematic review research. A comprehensive literature search was undertaken in the main electronic databases (PubMed (including Medline), Web of Science (Clarivate Analytics), Scopus (Elsevier), ScienceDirect (Elsevier), Cochrane Library (Wiley), and Wiley Online Library (Wiley)), limiting our search to English language documents published from 1990 onwards. A combination of free-text and thesaurus terms was used for concepts relevant to the topic. The search equations used are detailed in the Supplementary Materials. An automated alert for publication updates on all queried databases was created up to December 31, 2020.

Grey literature searches were undertaken using the health services research agencies.

**Figure 1:**
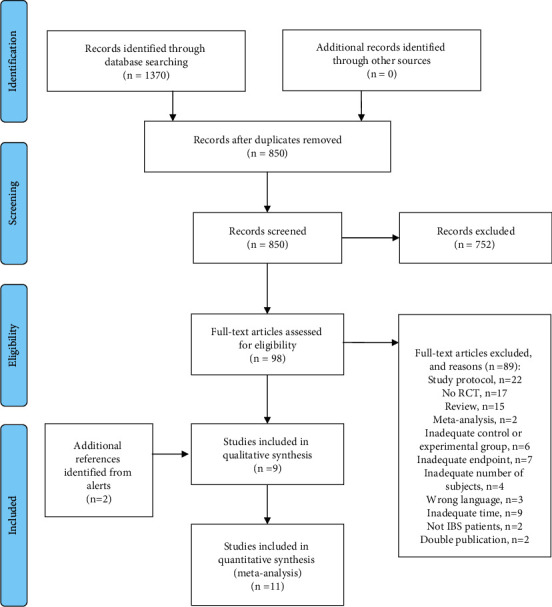
Preferred Reporting Items for Systematic Reviews and Meta-Analyses (PRISMA) flowchart of literature search for non-pharmacological interventions and irritable bowel syndrome.

PROSPERO and ClinicalTrials.gov were also searched, to ensure completeness, as recommended for systematic reviews. Two independent reviewers (F.A. and S.S.) examined all bibliographic records identified for title/abstract and then for full text. Any inconsistencies between reviewers were resolved by consensus with a third reviewer (F.M.). The study flowchart and reasons for exclusion of full-text articles are detailed in the PRISMA study flowchart (see Figure 1).

Data were extracted into an Excel file using a standardised Data Extraction Form (Cochrane Collaboration). Two reviewers (F.A. and S.S.) independently extracted data on the studies, including study design, the nature of the interventions, and the clinical outcomes. For each study, the main characteristics of the study population were also extracted. Any disagreements between the two reviewers were resolved by consensus.

The methodological quality of included studies was assessed independently by two of the authors (F.A and S.S) using the Cochrane risk of bias (RoB) tool version 2 for randomized trials [37]. Bias was assessed in five distinct areas. In each domain, one or more questions were answered, and answers led to a judgment of “low risk of bias,”, “of concern,” or “high risk of bias.” The results from each domain yielded an overall judgment of the risk of bias for the outcome being assessed. Any discrepancies were resolved by consensus with a third reviewer (F.M.).

Treatment effect was assessed using mean differences due to a mixture of change-from-baseline and postintervention value scores. Responder definitions differed between studies, precluding any estimate of effect measure from dichotomous data. Since many standard deviations for change were missing, attempts were made to contact the authors of the studies. As these data could not be obtained, imputation methods were used as recommended by the Cochrane group [38]. A method was used to impute missing standard deviations using an imputed value for the correlation coefficient (Corr) in the case where the baseline and final standard deviations were known. For the studies by Flick [39], Lowe [40], and MacPherson [41], which used the IBS-symptom severity score (IBS-SSS) questionnaire, the Corr value was imputed using data obtained at 3-month follow-up from the study by Piche [42], not included in the review. At 6-month follow-up, for the study by MacPherson [41], the Corr value was imputed from data from the study by Pei [43].

The characteristics of the studies, the population, and the results are summarized in a narrative manner and using summary tables.

Data were analysed using RevMan 5.4 statistical software. Statistical heterogeneity was tested using the Chi^2^ test and the *I*^2^ statistic. The *I*^2^ statistic describes the variability in treatment effect estimates due to heterogeneity. Because statistical heterogeneity was high (*I*^2^ > 50% or *p* < 0.01), a random-effects model was chosen to pool our data. Due to the number of studies (<10), we were not able to undertake statistical tests for small-study effects [44].

Given the substantial number of imputed data, meta-analysis was chosen on an exploratory basis.

The strength of evidence was assessed using a systematic scale and degrees of recommendation based on Oxford levels of evidence were used [45].

## 3. Results

Of 1370 records identified and screened at title/abstract level, 98 were examined for full text, of which 9 studies plus 2 additional studies identified via the database alert system were included, totalling 1590 participants (see Figure 1). Risks of bias and forest plots not shown in the main manuscript are included in the Supplementary Materials (see Figures S1–S12).

### 3.1. Study Types

The 11 studies were parallel-group RCTs, of which four evaluated the effectiveness of hypnotherapy [39, 46–48], and seven evaluated body-directed therapies: acupuncture [40, 41, 43, 49], osteopathic medicine [50], auriculotherapy [51], and reflexology [52] (Table 1). A sham control group was used in four body-directed therapy studies [40, 49, 51, 52], while for the seven others, the control group was SMT (National Health Service (NHS) lifestyle, drugs, and extra fibres) or SMT associated with various antistress interventions (group educational support therapy, attentive listening, and home exercise), in order to obtain the same duration of interaction with therapists as in the intervention groups.

In hypnotherapy studies, the therapists in the experimental group were psychologists or nurses qualified in hypnotherapy and those in the control group could be physicians or non-physicians (nurse, psychologists' assistants, dietician, and physiotherapist) [39, 47].

In acupuncture studies, the therapists in the experimental and sham therapy groups were identical [40, 49].

Four physicians provided reflexology, and the sham intervention consisted in applying pressure on nonreflex zones with the same number of contact sessions as in the active group [52].

In the osteopathic medicine study, an individualized treatment was performed by a single osteopath, while the SMT in the control group was provided by physicians.

### 3.2. Characteristics of Included Subjects

The patients included were refractory to medication and diet (Table 1).

The average number of patients included in the experimental groups was 67, and 37 in the control groups. Three studies had a large number of subjects (not less than 233) [39, 41, 43]. The mean age of participants was 33.2 years (range 13.3–42) for hypnotherapy, 43.9 years (range 42.7–46.4) for acupuncture, 48 years for reflexology, 15.4 years for auriculotherapy, and 42.8 years for osteopathic medicine. The proportion of women ranged from 44.8% to 90%. Two studies investigated the subtypes of IBS [43, 51]. Six studies specified IBS duration before inclusion, with participants who had suffered from IBS for at least 6 weeks [46], 3 months [49], 6 months [43], 12 months [48, 52], 13 years [41], and 15 years [52]. Three studies reported the severity of symptoms (moderate severity) [41, 43, 50].

### 3.3. Methodological Quality of Studies

Four studies had a high risk of bias [40, 46, 47, 52], three studies had a risk of bias considered to be of concern [41, 43, 50], and four studies had a low risk of bias [39, 48, 49, 51] (Figures S1–S6). All but one study [52] reported the use of a random component in the sequence generation process. Three of the four studies at high risk of bias did not assess blinded results [40, 46, 47]. The lack of double blinding accounted for the presence of high-performance bias in most trials [39, 41, 43, 46, 47, 48, 50]. In three trials [40, 49, 52], only advanced patients were blinded, and in only one trial [51] was double blinding possible.

### 3.4. Effectiveness of NPIs on IBS Symptoms

#### 3.4.1. Effectiveness of Body-Directed Therapies

Two studies used the IBS-SSS questionnaire to obtain an overall symptom score [41, 43]. Lowe et al. used a modified version of the Bowel Disease Questionnaire [40]. Three other studies used a Likert scale to obtain an overall symptom score [49, 50] or an abdominal pain intensity score [52]. Krasaelap et al. used the Pain-Frequency-Severity-Duration questionnaire to obtain a pain score [51] (Table 2).

Among six studies that evaluated the effectiveness of body-directed therapy at 3 months, only MacPherson et al. observed a significant improvement in overall IBS symptoms in patients under the intervention conditions (acupuncture) compared to SMT [41]. Hundscheid et al. showed no significant difference between osteopathic medicine and SMT [50]. Lowe et al. and Forbes et al. found no significant difference in overall IBS symptoms between acupuncture and sham procedures [40, 49]. A similar trend was observed between auriculotherapy or reflexology and sham procedures for abdominal pain [51, 52].

All the studies that evaluated the effectiveness of body-directed therapies at 6 months found a beneficial effect in overall IBS symptoms compared to SMT [41, 43, 50]. This effect was not sustained at 12-month follow-up [41].

#### 3.4.2. Effectiveness of Hypnotherapy

One study used the IBS-SSS questionnaire to obtain an overall symptom score [39]. One study used a Likert scale to obtain an abdominal pain intensity score [48]. Lindfors et al. used the Gastro Intestinal Severity questionnaire [47]. This questionnaire used a 7-point Likert scale to obtain an overall symptom score. Roberts et al. used a three-dimensional questionnaire (pain, constipation, and diarrhea) to obtain a score for each of these symptoms and an overall score [46].

Two of the three studies that evaluated the effectiveness of hypnotherapy at 3 months showed a significant improvement in overall symptoms or abdominal pain under the intervention conditions compared to SMT and supportive therapy [46, 47]. Flick et al. found no significant difference [39].

Among the three studies evaluating the effectiveness of hypnotherapy at 12 months, Vlieger et al. found a beneficial effect of hypnotherapy on abdominal pain compared to the association of SMT and supportive therapy [48]. Flick et al. and Roberts et al. found no significant difference in overall IBS symptoms or pain [39, 46].

### 3.5. Adverse Events

None of the RCTs reported serious adverse events, and only some (acupuncture and osteopathic medicine) reported mild and transient adverse events, indicating that acupuncture, hypnotherapy, osteopathic medicine, and auriculotherapy appear to have an acceptable safety profile. The benefit/risk ratio was largely acceptable for acupuncture and osteopathic medicine.

### 3.6. Exploratory Meta-Analyses

At three-month follow-up, there was a benefit of NPIs in reducing overall IBS symptoms without reaching a statistical difference (MD = −3.79; 95% CI −8.32 to 0.73 (see Figure S7)), with a major degree of heterogeneity across studies (*I*^2^ = 85%, *p* < 0.0001, *n* = 5 studies (see Figure S7)). The exclusion of outliers [41] yielded a lower but more accurate estimate of the treatment effect (MD = −0.57, 95% CI: −2.85 to 1.70 (see Figure S8)), still with substantial heterogeneity (*I*^2^ = 51%, *p*=0.11, *n* = 4 studies (see Figure S8)).

There was a nonsignificant benefit of body-directed therapies over SMT and sham procedures (MD = −3.02, 95% CI: −7.56 to 1.53 (see Figure S7)), but with very high heterogeneity (*I*^2^ = 87%, *p* < 0.0001, *n* = 4 studies (see Figure S7)). One study [41] was an outlier, and its exclusion reduced the treatment effect (MD = −0.28, 95% CI −2.14 to 1.59 (see Figure S8)) and the level of heterogeneity (*I*^2^ = 40%, *p*=0.19, *n* = 3 studies (see Figure S8)). The benefit of mind-body therapy was nonsignificant compared with supportive therapy (MD = −14.10, 95% CI −30.43 to 2.23 (see Figure S7)).

There were subgroup differences according to the type of control (*I*^2^ = 62.4%, *p*=0.10 (see Figure S9)), treatment duration (*I*^2^ = 88.2%, *p*=0.004 (see Figure S10)), and the instrument used to measure outcomes (*I*^2^ = 86.3%, *p*=0.007 (see Figure S11)). However, there was a high level of heterogeneity within each of these subgroups (*I*^2^ > 50%), except in the sham therapy group and in the group with a treatment duration of 30 minutes, where the trials were homogeneous (*I*^2^ = 0%, *p*=0.82, *n* = 2 studies (see Figures S9 and S10)).

At 6 months, there was a significant benefit of acupuncture [41, 43] compared to SMT for overall IBS symptoms (MD = −33.56, 95% CI −46.13 to −21.00 (see Figure S12)), with a low level of heterogeneity (*I*^2^ = 26%, *p*=0.24, *n* = 2 studies (see Figure S12)).

### 3.7. Levels of Evidence and Grade of Recommendation

All included studies were assessed as reporting evidence levels 2b with the exception of four studies with evidence level 1b [39, 48, 49, 51] (Table 3). On the basis of these Oxford levels of evidence, we gave grade B recommendations for body-directed therapies and grade C for mind-body therapies, taking into account the quality of the studies, the heterogeneity of effect sizes, and the safety characteristics.

## 4. Discussion

In this systematic review, 11 studies were included totalling 1590 adult and pediatric patients. All studies were assessed for methodological quality, and all patient clinical data and specific trial characteristics were described and analysed. In addition, an exploratory meta-analysis was performed, including subgroup and sensitivity analyses to search for interactions between different covariates and the treatment effect.

To explore possible sources of heterogeneity, sensitivity analyses were performed excluding outliers, and this showed no significant change in the pooled results, particularly in terms of change in statistical significance. In addition, the subgroup analyses showed no interactions between the treatment effect and the factors defining the subgroups (treatment duration, type of control, and outcome measurement tools).

It has been found that body-directed therapies can reduce the intensity of overall IBS symptoms compared to SMT [41, 43, 50]. However, none of the studies evaluating the effectiveness of body-directed therapy compared to sham procedures showed a significant effect [40, 49, 51, 52].

Evaluation of the effectiveness of hypnotherapy showed discordant results for overall IBS symptoms and abdominal pain.

Our exploratory meta-analysis reported similar results to a recent network meta-analysis [53] regarding the effectiveness and safety of non-pharmacological interventions for IBS symptoms. Although the scope of this paper was different from our study (it included also diet modifications and dietary complements such as probiotics), this study confirmed the potential effectiveness of acupuncture. Thus, the results from these two meta-analyses complement each other.

Based on the levels of evidence from the studies included in our systematic review, we consider that body-directed therapies, namely, osteopathic medicine and acupuncture, have provided acceptable evidence of benefit for IBS symptoms.

It is interesting to note that the design of the included trials can have an impact on the observed results. In particular, it was found that the type of control group, namely, SMT or supportive therapy, versus sham therapies may influence trials outcomes. This observation is consistent with a recent systematic review that recommended using an optimal placebo as a control, defined as an experience comparable to the intervention. In the case of an inadequate placebo, the response would be more favorable to the intervention [35]. However, choosing the adequate sham therapy, especially in the field of body-directed therapies, is a difficult task. This is illustrated in the RCT of auriculotherapy [51], where the control group received a sham therapy (inactive medical device that was identical to the active device, without electric current application). The active device used neurostimulation below the threshold of perception, in order to obtain a similar feeling to the patient as the inactive device. However, 75% of subjects in each group accurately identified their device as sham or active. Despite the fact that patients were clearly able to differentiate between the active and sham treatment, the RCT did not demonstrate any positive effect of the active treatment.

On the basis of the results, it cannot be ruled out that sham therapies, especially in the field of body-directed interventions, may produce an effective therapy distinct from the placebo effect, which would impair the demonstration of a positive effect of the active arm of the RCT.

Waiting list may be an option to establish control groups in the setting of NPI studies. However, this type of control group was not used in the studies included in this review. According to Cunningham et al. [54], such a group may overestimate the size of the treatment effect because the subjects included in the waiting list would tend not to commit to change for the better because they are in a situation where they are awaiting treatment. This waiting-list group may therefore have a smaller effect than that encountered in a standard care group.

The improvement of clinical trials requires the consideration of patient expectations as an important factor in the therapeutic outcome [54, 55]. Patient expectations may be influenced by the severity of baseline symptoms. This should further encourage future research to select patients with similar levels of symptom severity.

This review includes studies with questionnaires frequently reported in the literature, Likert scales, and IBS-SSS [39, 41, 43, 48, 50, 52]. This latter instrument has already been identified as having good reproducibility [56]. The results indicated that the treatment effect size did not vary according to the questionnaire used for overall IBS symptoms. Although with both questionnaires, the data obtained on overall symptoms incorporates quality of life, it is nonetheless important to assess the impact of NPIs on quality of life and other patient-reported outcomes using specific questionnaires, in order to properly define the disease experience. In addition, future studies could investigate whether there is a stronger correlation between changes on the IBS-SSS and changes in quality of life or psychological status, compared to Likert scales.

The study has several limitations. As expected, there was major heterogeneity across studies. This was not explained by the subgroup and sensitivity analyses and could be due to high random variability in the treatment effect, albeit without being able to link these fluctuations to specific factors. Due to the large amount of missing data, an exploratory meta-analysis was proposed whose results should be interpreted with caution. Not all trials in the review were included in the meta-analysis. Based on this synthesis of trial-related factors, including frequency of treatment and number of therapists, studies showed heterogeneous results at 3, 6, and 12 months of follow-up. Furthermore, due to the studies that were identified and their corresponding data, we have preferred not to conduct an indirect comparison of interventions due to the high potential for biased estimates.

In hypnotherapy trials, the number of subjects included was small with the exception of one trial that accrued 342 subjects. In acupuncture trials with sham procedures, the number of subjects was small, whereas sample sizes were larger in the acupuncture trials with SMT (519 and 233 subjects). The review includes small studies that may provide information that is untypical of the treatment effects usually observed.

Another limitation is the risk of bias for a large proportion of the RCTs, rated as high or moderate, which could reduce confidence in the results.

## 5. Conclusions

Existing literature suggests that mind-body therapies may have potential in IBS. Conversely, the findings of this review support the potential effectiveness of body-directed therapies (acupuncture and osteopathic medicine) on overall IBS symptoms at 6 months of follow-up. From published comparative studies, the effect of hypnotherapy is controversial. The review demonstrates that, to date, there is insufficient evidence to identify and define an optimal sham therapy. Based on the limitations identified, future research based on trials of better methodological quality should be undertaken, ensuring that a large number of subjects with similar baseline symptom severity are included. Further efforts should be undertaken to improve the design of clinical trials in IBS, in particular to identify an optimal control group to clarify the impact of NPIs, including osteopathic medicine, on IBS.

## Figures and Tables

**Table 1:** Description of the 11 randomized clinical trials included and patients.

Year	Authors	Country location	Design	NPI	Control	IBS definition	IBS duration	IBS severity	Numbers of patients		Mean age (y)		Female (%)		Frequency and duration of treatment	Follow-up	Drugs	Adverse events
									NPI	C.	NPI	C.	NPI	C.				
2012	MacPherson et al. [41]	UK	RCT	Acupuncture (+standard care)	Standard care	Refractory	13 years	Moderate (100 or more)	116	117	44.3	42.7	82.0	79.5	10 weekly individualised sessions 45 min	12 months	NA	Pain
2005	Forbes et al. [49]	UK	RCT single-blind	Acupuncture	Sham	Refractory Rome II Manning	Since at least 3 months	NA	27	32	43.0	44.4	59.2	71.9	10 weekly individualised sessions 30 min	13 weeks	CT (psy. dr.)	Bruise, nervous fatigue
2017	Lowe et al. [40]	Canada	RCT single-blind	Acupuncture	Sham	Refractory Rome I	NA	NA	43	36	42.0	43.0	84.0	72.0	2-3 times per week for 1 month. 30 min	12 weeks	CT	NA
2020	Pei et al. [43]	USA	RCT	Acupuncture	Standard care	Refractory Rome III	Since 6 months or more	Moderate (75)	344	175	45.9	47.0	48.2	49.7	2-3 times per week for 1.5 months. 30 min	18 weeks	Emergency medicines	Hematoma and pain
2012	Lindfors study 1 [47]	Sweden	RCT	Hypnotherapy	Standard care	Refractory Rome II	NA	NA	45	45	43	41	77.7	80.0	12 weekly individualised sessions. 60 min	12 weeks	CT	No
2006	Roberts et al. [46]	UK	RCT	Hypnotherapy (+standard care)	Standard care	Refractory Rome II	Since over 6 weeks	NA	40	41	42.4	40.8	80.0	90.2	Five half-hour sessions spaced about a week apart	12 months	CT	NA
2007	Vlieger et al. [48]	The Netherlands	RCT	Hypnotherapy	Standard care	Functional abdominal pain. Refractory Rome II	Since at least 12 months	NA	27	25	13.2	13.4	67.0	84.0	Six fifty-minute sessions over a three-month period	12 months	NA	NA
2019	Flick et al. [39]	The Netherlands	RCT	Hypnotherapy	Standard care	Refractory Rome III	NA	NA	300	54	37.3	34.5	76.5	89.0	Six individual or group hypnotherapy sessions every 2 weeks. 60 min	12 months	CT	No
2020	Krasae-lap et al. [51]	USA	RCT double-blind	Auriculotherapy	Sham	Refractory Rome III	NA	NA	27	23	15.3	15.6	24.0	21.0	Active or simulated stimulation for 5 days/week with 2 days off per week for a total of 4 weeks	12 weeks	NA	No
2002	Tovey et al. [52]	UK	RCT single-blind	Reflexology	Sham	Refractory Rome	From 18 months to 15 y	NA	19	15	48		83.35		Reflexology group. Six treatments of 30 min (4 per week and two every 15 days)	12 weeks	NA	NA
2007	Hund-scheid et al. [50]	The Netherlands	RCT	Osteopathic medicine	Standard care	Refractory Rome II	NA	Moderate (37 to 110)	20	19	46.5	41	70.0	47.36	5 sessions once every 2 or 3 weeks 30 min	6 months	No	No

RCT: randomized controlled trial; NPI: non-pharmacological intervention; C.: control; CT: continuation of usual treatment; NA: not available.

**Table 2:** Description of the symptom outcomes and main results of the 11 RCTs.

Authors	NPI	Instrument	Outcomes
MacPherson et al. [41]	Acupuncture (+SC)	IBS-SSS	*Overall IBS Symptoms*. Superiority of acupuncture over antispasmodics, antidiarrheals and laxatives at 3-month and 6-month follow-up (*p* < 0.05). No significant differences were seen at 12-month follow-up
Forbes et al. [49]	Acupuncture	Likert	*Overall IBS Symptoms.* No significant differences were seen between the acupuncture group and the sham procedures group (false acupuncture points) at 3-month follow-up
Lowe et al. [40]	Acupuncture	BDQ	*Overall IBS Symptoms*. No significant differences were seen between the acupuncture group and the sham procedures group (false acupuncture points) at 3-month follow-up
Pei et al. [43]	Acupuncture	IBS-SSS	*Overall IBS Symptoms.* Superiority of acupuncture over antidiarrheals and laxatives at 6-month follow-up (*p* < .05)
Lindfors et al. [47]	Hypnotherapy	GISQ	*Overall IBS Symptoms.* Superiority of hypnotherapy over supportive therapy at 3-month follow-up (*p* < .05)
Roberts et al. [46]	Hypnotherapy	SS3D	*Overall IBS Symptoms* *+* *Abdominal Pain.* Superiority of hypnotherapy over SMT at 3-month follow-up (*p* < .05). No significant differences were seen at 12-month follow-up
Vlieger et al. [48]	Hypnotherapy	Likert	*Abdominal Pain.* Superiority of hypnotherapy over association SMT/supportive therapy at 12-month follow-up (*p* < .05)
Flick et al. [39]	Hypnotherapy	IBS-SSS	*Overall IBS Symptoms*. No significant differences were seen between the hypnotherapy group and the control group (supportive therapy) at 3-month and 12-month follow-up
Krasaelap et al. [51]	Auriculotherapy	PFSD	*Abdominal Pain.* No significant differences were seen between the auriculotherapy group and the sham procedures group at 3-month follow-up
Tovey et al. [52]	Reflexology	Likert	*Abdominal Pain.* No significant differences were seen between the reflexology group and the sham procedures group (massage on nontherapeutic points) at 3-month follow-up
Hundscheid et al. [50]	Osteopathic medicine	Likert	*Overall IBS Symptoms.* No significant differences were seen between the osteopathic medicine group and the control group (SMT) at 3-month follow-up. Superiority of osteopathic medicine over SMT at 6-month follow-up (*p* < 0.05)

SC: standard care; BDQ: Bowel Disease Questionnaire; IBS-SSS: Irritable Bowel Syndrome Symptom Severity Score; GISQ: Gatrointestinal Severity Questionnaire; SS3D: Symptom Score 3 Dimensions: Pain, Diarrhea, Constipation; PFSD: Pain-Frequency-Severity-Duration worst pain score.

**Table 3:** Evaluation of levels of evidence and recommendations of included studies according to the Centre for Evidence-Based Medicine (CEBM), Oxford.

NPIs	Authors	Levels of evidence from the CEBM, Oxford (b)	Grade of recommendation per CEBM, Oxford
Body-directed therapies	MacPherson et al. [41]	2	B
	Forbes et al. [49]	1	
	Lowe et al. [40]	2	
	Pei et al. [43]	2	
	Krasaelap et al. [51]	1	
	Tovey et al. [52]	2	
	Hundscheid et al. [50]	2	

Mind-body therapies	Lindfors et al. [47]	2	C
	Roberts et al. [46]	2	
	Vlieger et al. [48]	1	
	Flick et al. [39]	1	

NPIs: non-pharmacological interventions, 1b = individual RCT, 2b = individual cohort study (including low-quality RCT), B = consistent level 2 or 3 studies or extrapolations from level 1 studies, and C = level 4 studies or extrapolations from level 2 or 3 studies.

## Supplementary Materials

Supplementary Materials Literature search strategies (September 2019). Figure S1: risk of bias in the reviewed hypnotherapy studies, as measured by Rob 2.0. Figure S2: risk of bias in the reviewed acupuncture studies, as measured by Rob 2.0. Figure S3: risk of bias in the reviewed auriculotherapy study, as measured by Rob 2.0. Figure S4: risk of bias in the reviewed reflexology study, as measured by Rob 2.0. Figure S5: risk of bias in the reviewed osteopathic medicine studies, as measured by Rob 2.0. Figure S6: “risk of bias” graph: review authors' judgements about each “risk of bias” domain. Figure S7: forest plot of studies of NPIs versus sham or SMT or supportive therapy on overall IBS symptoms by type of therapy at 3 months. Figure S8: forest plot of studies of NPIs versus sham or SMT or supportive therapy on overall IBS symptoms by type of therapy at 3 months (after exclusion of outliers). Figure S9: forest plot of studies of NPIs vs. sham or SMT or supportive therapy on overall IBS symptoms by type of control group at 3 months. Figure S10: forest plot of studies of NPIs versus sham or SMT or supportive therapy on overall IBS symptoms by treatment duration at 3 months. Figure S11: forest plot of studies of NPIs versus sham or SMT or supportive therapy on overall IBS symptoms by instrument at 3 months. Figure S12: forest plot of studies of body-directed therapies vs. SMT on overall IBS symptoms at 6 months.
