# Supplementary material for: Effectiveness of Non-Pharmacological Interventions for Irritable Bowel Syndrome: A Systematic Review
Source: Evid Based Complement Alternat Med. 2021 Nov 8;2021:4404185. doi: 10.1155/2021/4404185 (PMC8592737; doi:10.1155/2021/4404185)
Supplement: Supplementary Materials — Literature search strategies (September 2019). Figure S1: risk of bias in the reviewed hypnotherapy studies, as measured by Rob 2.0. Figure S2: risk of bias in the reviewed acupuncture studies, as measured by Rob 2.0. Figure S3: risk of bias in the reviewed auriculotherapy study, as measured by Rob 2.0. Figure S4: risk of bias in the reviewed reflexology study, as measured by Rob 2.0. Figure S5: risk of bias in the reviewed osteopathic medicine studies, as measured by Rob 2.0. Figure S6: “risk of bias” graph: review authors' judgements about each “risk of bias” domain. Figure S7: forest plot of studies of NPIs versus sham or SMT or supportive therapy on overall IBS symptoms by type of therapy at 3 months. Figure S8: forest plot of studies of NPIs versus sham or SMT or supportive therapy on overall IBS symptoms by type of therapy at 3 months (after exclusion of outliers). Figure S9: forest plot of studies of NPIs vs. sham or SMT or supportive therapy on overall IBS symptoms by type of control group at 3 months. Figure S10: forest plot of studies of NPIs versus sham or SMT or supportive therapy on overall IBS symptoms by treatment duration at 3 months. Figure S11: forest plot of studies of NPIs versus sham or SMT or supportive therapy on overall IBS symptoms by instrument at 3 months. Figure S12: forest plot of studies of body-directed therapies vs. SMT on overall IBS symptoms at 6 months. [file 4404185.f1.docx]

**Literature search strategies (September 2019)**

**1/Pubmed [including Medline]: 356 results**

(("Complementary Therapies"[Mesh:noexp] OR "Osteopathic Medicine"[Mesh] OR "Manipulation, Osteopathic"[Mesh] OR "Acupuncture Therapy"[Mesh] OR "Acupuncture"[Mesh] OR "Hypnosis"[Mesh] OR "Chiropractic"[Mesh] OR "Auriculotherapy"[Mesh] OR "Musculoskeletal Manipulations"[Mesh] OR "Reflexotherapy"[Mesh] AND ("Gastrointestinal Diseases"[Mesh:noexp] OR "Colonic Diseases, Functional"[Mesh] OR "Irritable Bowel Syndrome"[Mesh]) AND ("Evaluation Studies as Topic"[Mesh] OR "Meta-Analysis as Topic"[Mesh] OR "Treatment Outcome"[Mesh] OR "Clinical Trials as Topic"[Mesh] OR "Pragmatic Clinical Trials as Topic"[Mesh] OR "Controlled Clinical Trials as Topic"[Mesh] OR "Adaptive Clinical Trials as Topic"[Mesh] OR "Outcome Assessment (Health Care)"[Mesh] OR "Systematic Reviews as Topic"[Mesh] OR "Multicenter Studies as Topic"[Mesh] OR "Guidelines as Topic"[Mesh] OR "Randomized Controlled Trials as Topic"[Mesh] OR "Review Literature as Topic"[Mesh] OR "Research Design"[Mesh] OR "Practice Guidelines as Topic"[Mesh] OR "Validation Studies as Topic"[Mesh] OR "Consensus Development Conferences as Topic"[Mesh] OR "Consensus"[Mesh] OR "Bayes Theorem"[Mesh] OR "efficacy"[All fields] OR "evaluation"[All fields]) AND (("1990/01/01"[PDAT] : "2019/09/23"[PDAT]) AND English[lang])) OR (("Osteopathic"[Tiab] OR "Acupuncture"[Tiab] OR "Hypnosis"[Tiab] OR "Hypnoses"[Tiab] OR "Hypnotherapy"[Tiab] OR "Hypnotherapies"[Tiab] OR "Chiropractic"[Tiab] OR "Auriculotherapy"[Tiab] OR "Reflexotherapy"[Tiab] OR "non-drug treatments"[Tiab] OR "Manipulation"[Tiab] OR "Manipulations"[Tiab]) AND ("Functional Colonic Diseases"[Tiab] OR "Functional Colonic Disease"[Tiab] OR "Irritable Bowel Syndrome"[Tiab] OR "Irritable Colon"[Tiab]) AND ("efficacy"[Tiab] OR "evaluation"[Tiab] OR "Meta-Analysis"[Tiab] OR "Meta Analysis"[Tiab] OR "Clinical Trials"[Tiab] OR "Outcome Assessment"[Tiab] OR "Systematic Reviews"[Tiab] OR "Multicenter Studies"[Tiab] OR "Guidelines"[Tiab] OR "Randomized"[Tiab] OR "Review Literature"[Tiab] OR "Research Design"[Tiab] OR "Practice Guidelines"[Tiab] OR "Validation Studies"[Tiab] OR "Consensus"[Tiab] OR "Bayes Theorem"[Tiab]) AND (("1990/01/01"[PDAT] : "2019/09/23"[PDAT]) AND English[lang]))

**2/Web of Science [Clarivate Analytics]: 312 results**

**TOPIC:** (manipulati* OR osteopath* OR acupunct* OR hypnos* OR hypnotherap* OR chiropract* OR auriculotherap* OR reflexotherap* OR "non* drug treatment*") *AND* **TOPIC:** ("colonic disease*" OR "irritable bowel" OR "irritable colon") *AND* **TOPIC:** (effic* OR evaluati*) *AND* **LANGUAGE:** (English). **Timespan:** 1990-2019 **Indexes:** SCI-EXPANDED, SSCI, A&HCI, CPCI-S, CPCI-SSH, ESCI, CCR-EXPANDED, IC.

**3/Scopus [Elsevier]: 404 results**

(TITLE-ABS-KEY ((manipulati* OR osteopath* OR acupunct* OR hypnos* OR hypnotherap* OR chiropract* OR auriculotherap* OR reflexotherap* OR "non* drug treatment*")) AND TITLE-ABS-KEY (("colonic disease*" OR "irritable bowel" OR "irritable colon")) AND TITLE-ABS-KEY ((effic* OR evaluati*)) AND LANGUAGE (english)) AND PUBYEAR > 1989 AND PUBYEAR < 2020

**4/ ScienceDirect [Elsevier]: 131 results**

**Equation 1: 89 results**

Find articles with these terms : (efficacy OR evaluation) Year(s) : 1990-2019

Title, abstract or author-specified keywords : (manipulation OR osteopathy OR acupuncture OR hypnosis OR hypnotherapy OR chiropractic) AND ("colonic disease" OR "irritable bowel" OR "irritable colon")

**Equation 2:** **42 results**

Find articles with these terms : (efficacy OR evaluation) Year(s) : 1990-2019

Title, abstract or author-specified keywords : (auriculotherapy OR reflexotherapy OR "non-drug treatment") AND ("colonic disease" OR "irritable bowel" OR "irritable colon")

**5/Cochrane Library [Wiley]: 130 results (5 Cochrane Reviews + 125 Trials)**

**5 Cochrane Reviews** matching (manipulati* OR osteopath* OR acupunct* OR hypnos* OR hypnotherap* OR chiropract* OR auriculotherap* OR reflexotherap* OR "non* drug treatment*") in Title Abstract Keyword AND ("colonic disease*" OR "irritable bowel" OR "irritable colon") in Title Abstract Keyword AND (effic* OR evaluati*) in Title Abstract Keyword - with Cochrane Library publication date Between Jan 1990 and Sep 2019 (Word variations have been searched)

**125 Trials** matching (manipulati* OR osteopath* OR acupunct* OR hypnos* OR hypnotherap* OR chiropract* OR auriculotherap* OR reflexotherap* OR "non* drug treatment*") in Title Abstract Keyword AND ("colonic disease*" OR "irritable bowel" OR "irritable colon") in Title Abstract Keyword AND (effic* OR evaluati*) in Title Abstract Keyword - with Cochrane Library publication date Between Jan 1990 and Sep 2019 (Word variations have been searched)

**6/ Wiley Online Libray [Wiley]: 37 results**

**37results**

for"(manipulati* OR osteopath* OR acupunct* OR hypnos* OR hypnotherap* OR chiropract* OR auriculotherap* OR reflexotherap* OR "non* drug treatment*")" in Abstract and "("colonic disease*" OR "irritable bowel" OR "irritable colon")" in Abstract and "(effic* OR evaluati*)" in Abstract

Publication Date : 01/1990 to 09/2019

**0results** for"(manipulati*+OR+osteopath*+OR+acupunct*+OR+hypnos*+OR+hypnotherap*+OR+chiropract*+OR+auriculotherap*+OR+reflexotherap*+OR+"non*+drug+treatment*")" in Keywords and "("colonic disease*" OR "irritable bowel" OR "irritable colon")" in Keywords and "(effic* OR evaluati*)" in Keywords

Publication Date : 01/1990 to 09/2019

**0results** for"(manipulati*+OR+osteopath*+OR+acupunct*+OR+hypnos*+OR+hypnotherap*+OR+chiropract*+OR+auriculotherap*+OR+reflexotherap*+OR+"non*+drug+treatment*")" in Title and "("colonic disease*" OR "irritable bowel" OR "irritable colon")" in Title and "(effic* OR evaluati*)" in Title

Publication Date : 01/1990 to 09/2019

**Criteria Lindfors 2012 Roberts 2006 Vlieger 2007 Filck 2019**


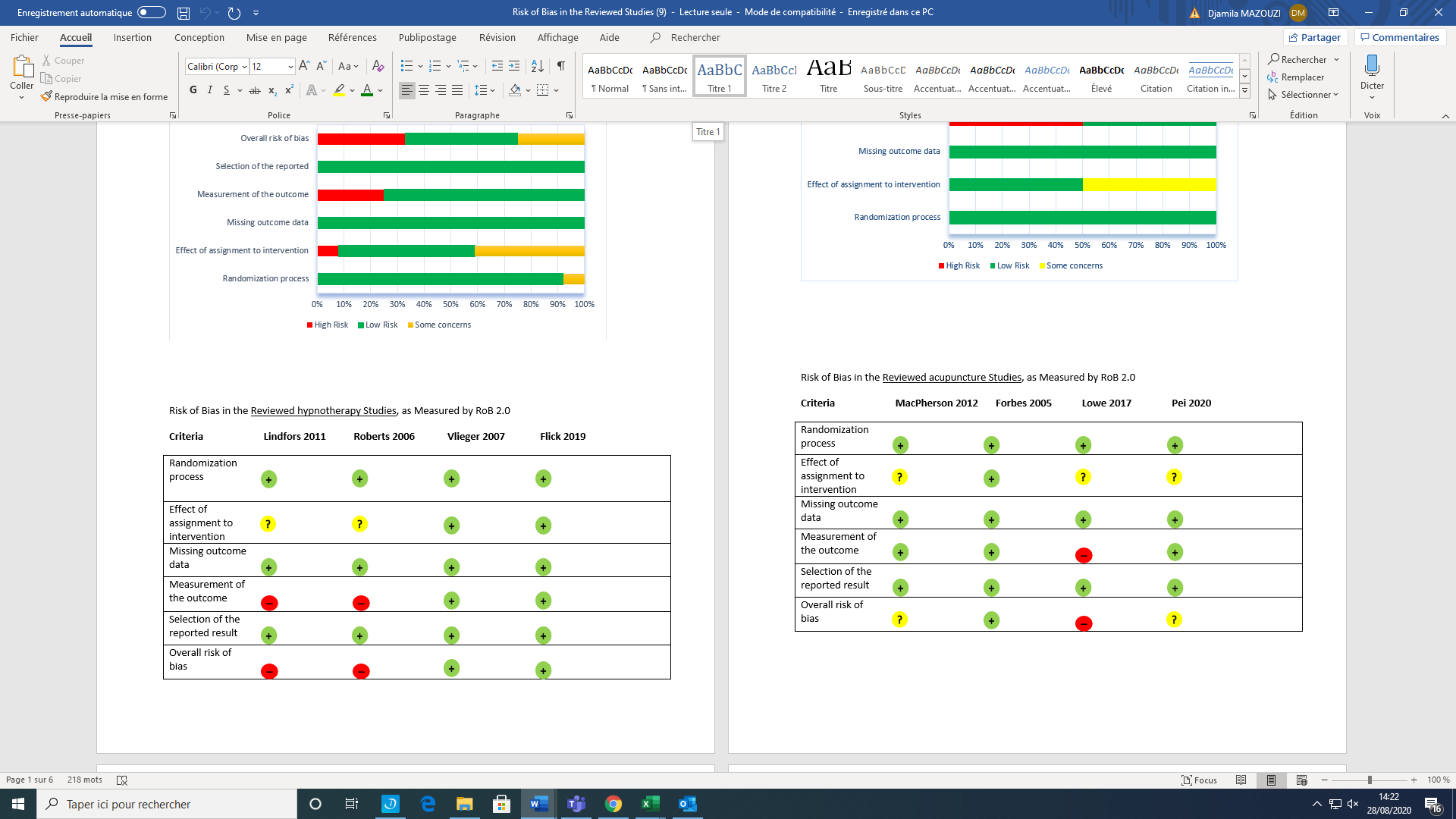


Figure S1: Risk of Bias in the Reviewed hypnotherapy Studies, as Measured by Rob 2.0

**Criteria MacPherson 2012 Forbes 2005 Lowe 2017 Pei 2020**


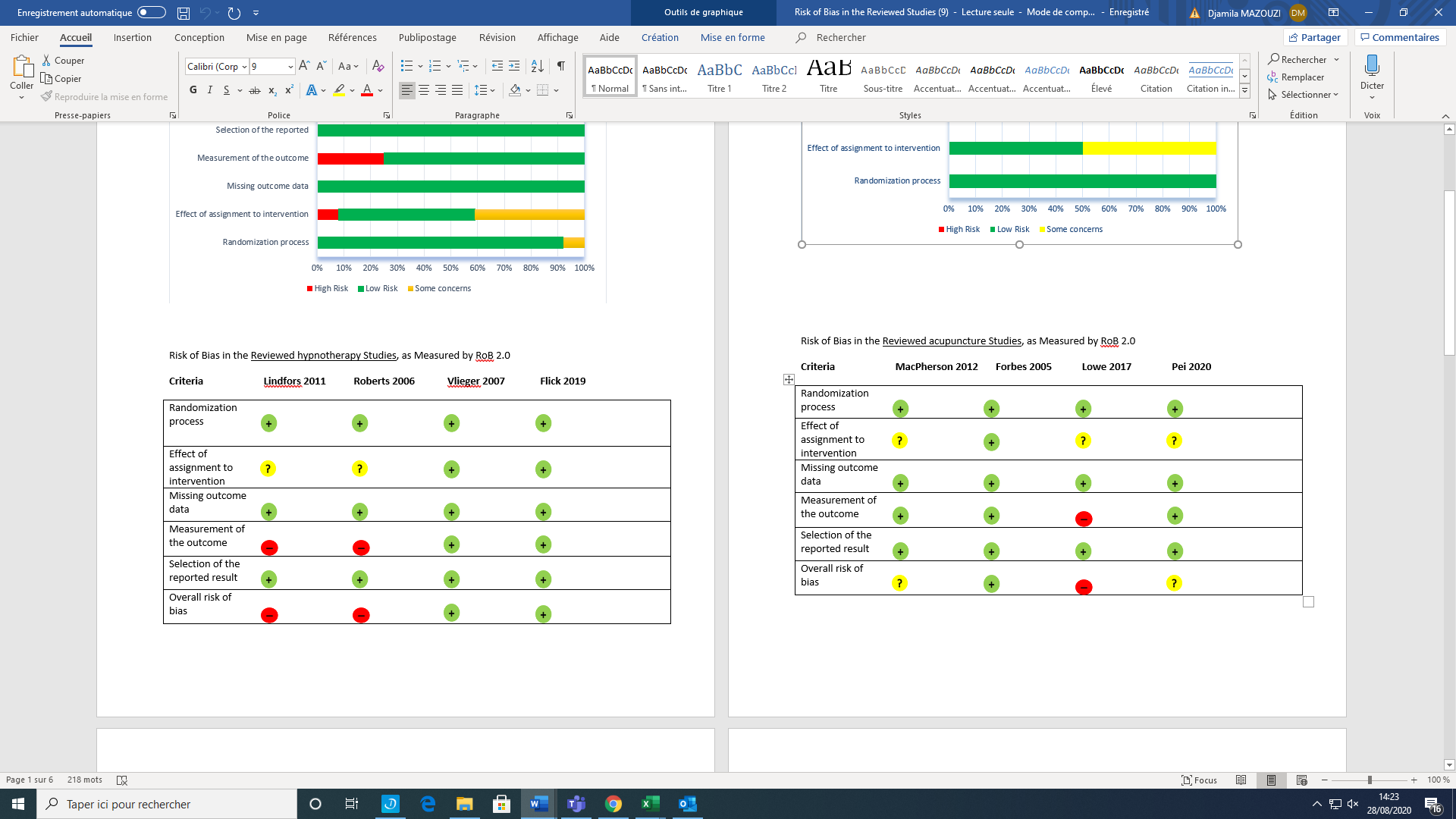


Figure S2: Risk of Bias in the Reviewed acupuncture Studies, as Measured by Rob 2.0

**Criteria Krasaelap 2019**


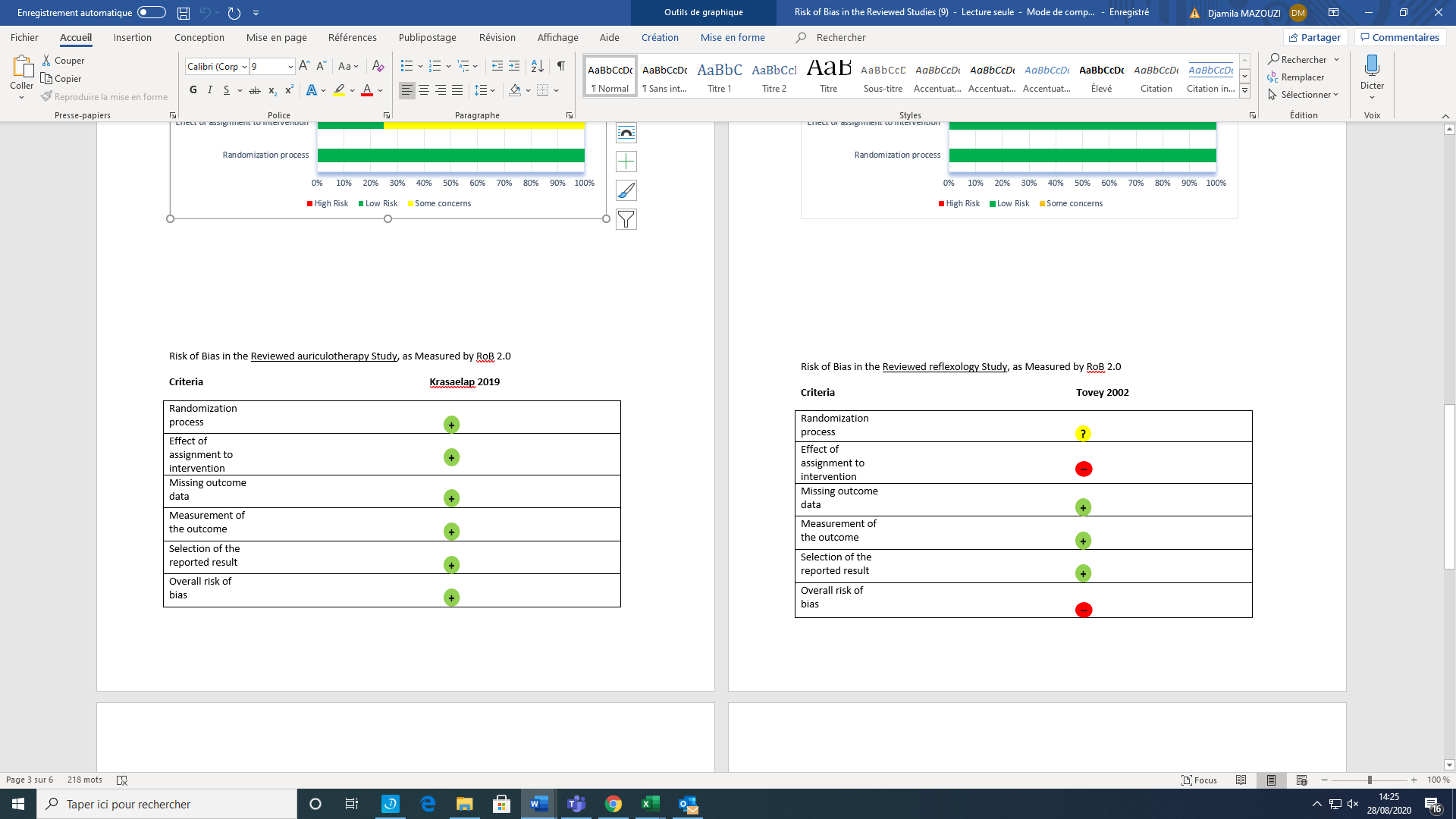


Figure S3: Risk of Bias in the Reviewed auriculotherapy Study, as Measured by Rob 2.0

**Criteria Tovey 2002**


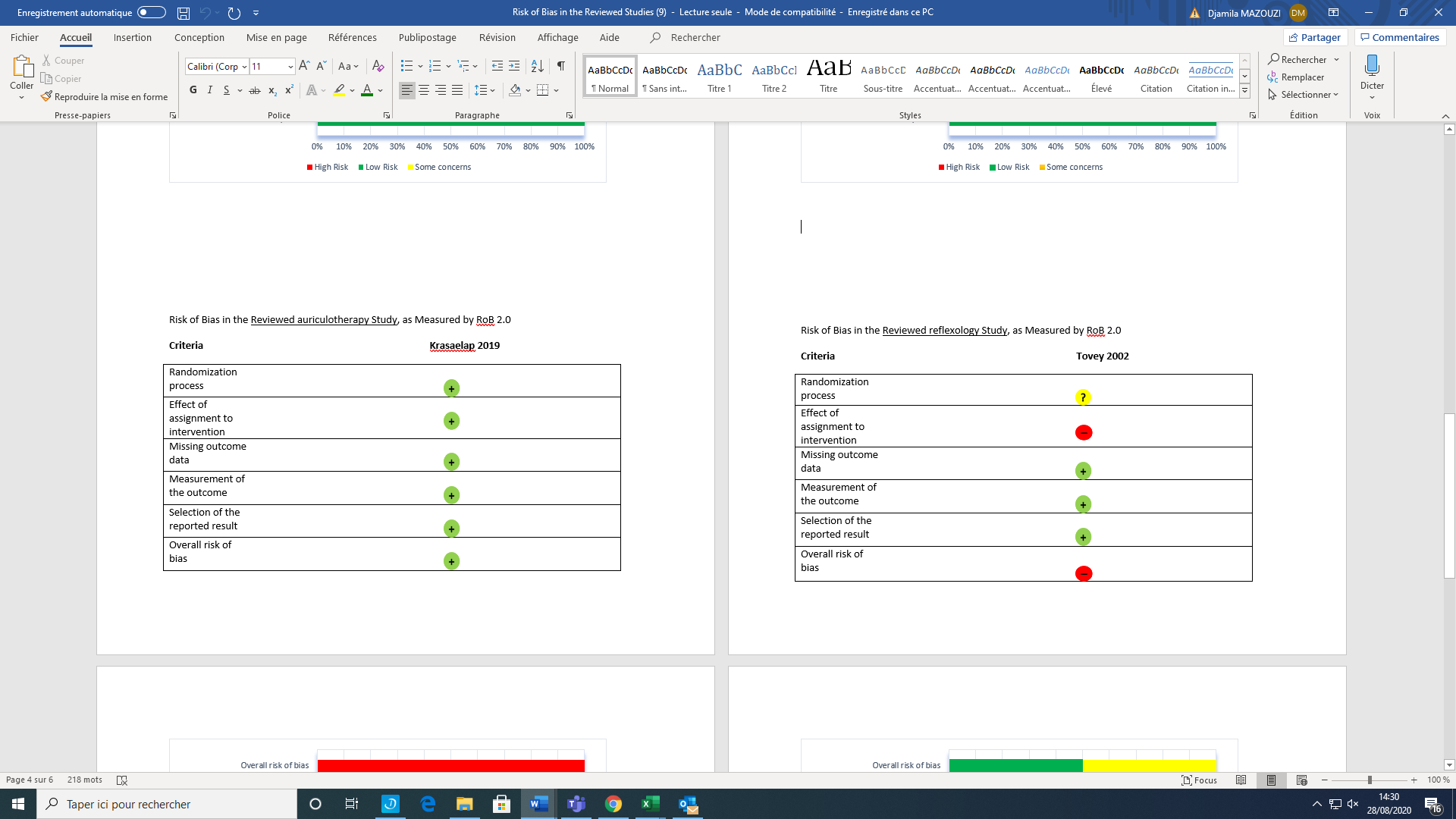


Figure S4: Risk of Bias in the Reviewed reflexology Study, as Measured by Rob 2.0

**Criteria Hundscheid 2007**


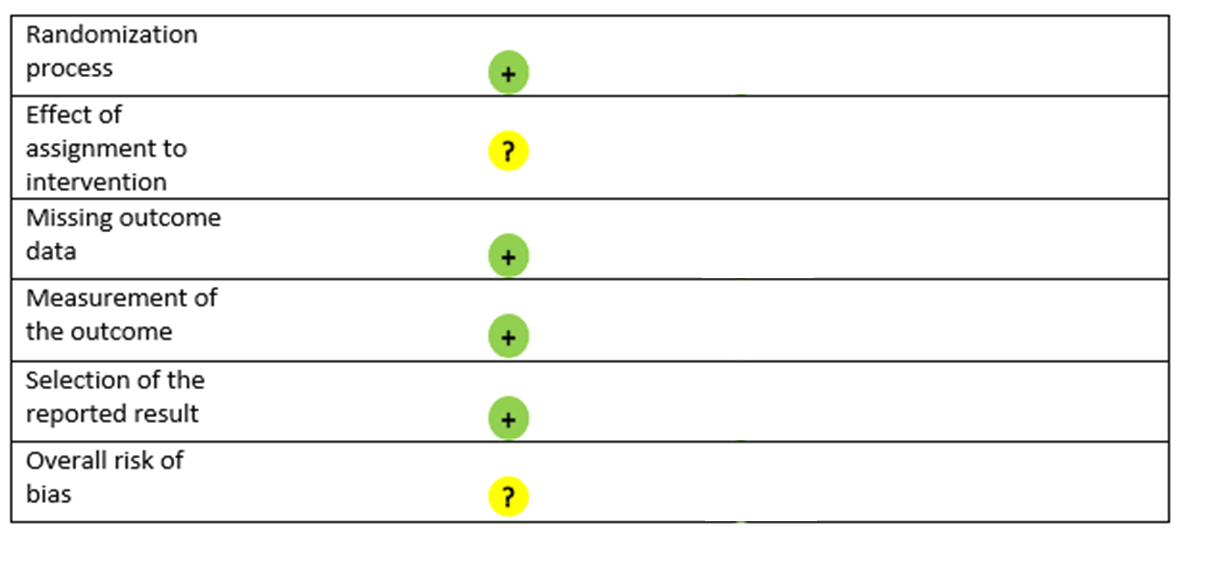


Figure S5: Risk of Bias in the Reviewed osteopathy Studies, as Measured by Rob 2.0


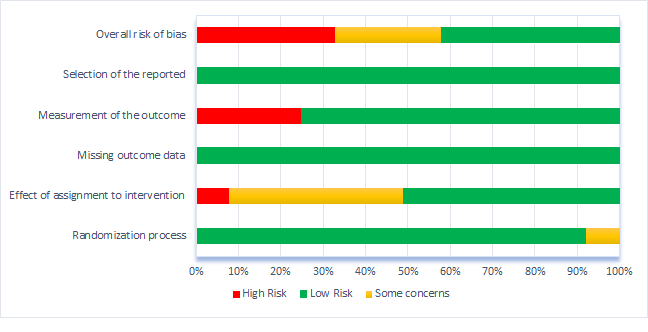


Figure S6: 'Risk of bias' graph: review authors' judgements about each 'risk of bias' domain


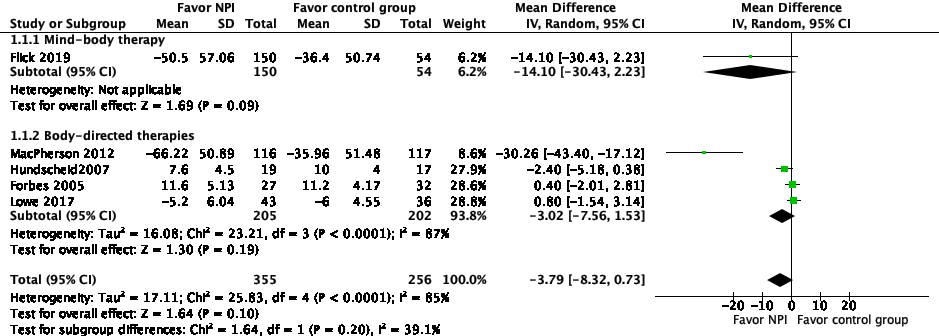


Figure S7: Forest plot of studies of NPIs vs sham or SMT or supportive therapy on overall IBS symptoms by type of therapy at 3 months

**
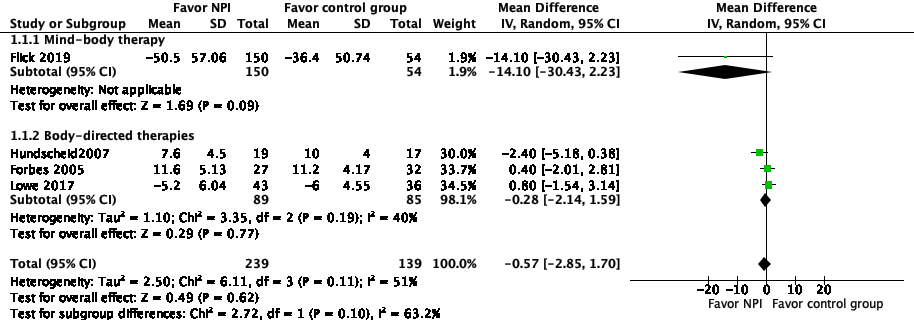
**

Figure S8: Forest plot of studies of NPIs vs sham or SMT or supportive therapy on overall IBS symptoms by type of therapy at 3 months (after exclusion of outliers)


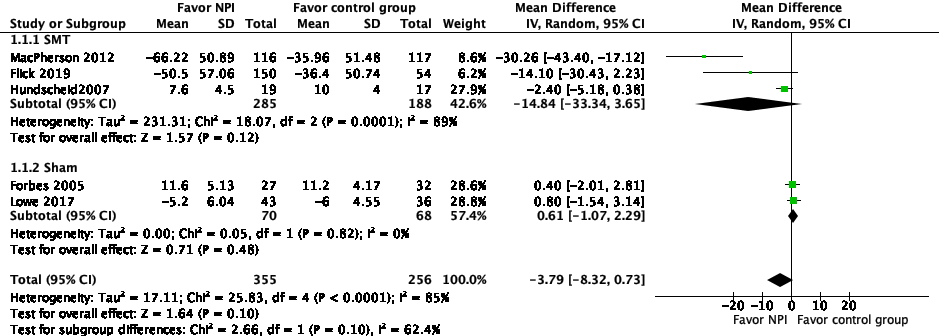


Figure S9: Forest plot of studies of NPIs vs sham or SMT or supportive therapy on overall IBS symptoms by type of control group at 3 months

**
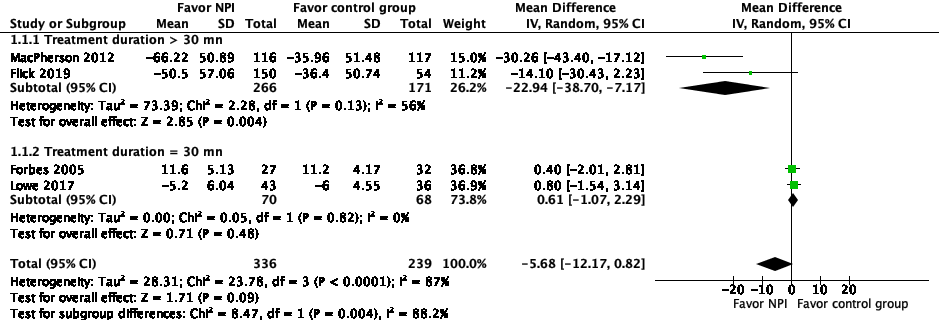
**

Figure S10: Forest plot of studies of NPIs vs sham or SMT or supportive therapy on overall IBS symptoms by treatment duration at 3 months

**
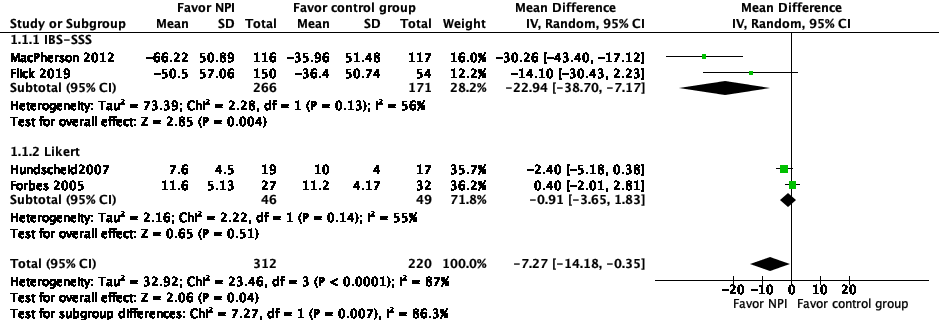
**

Figure S11: Forest plot of studies of NPIs vs sham or SMT or supportive therapy on overall IBS symptoms by instrument at 3 months


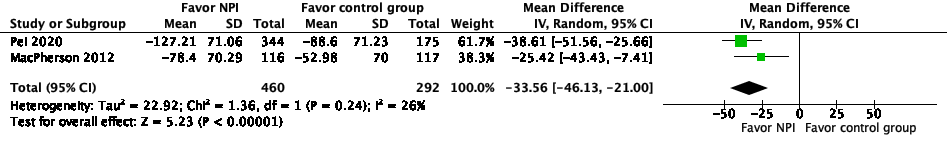


Figure S12: Forest plot of studies of body-directed therapies vs SMT on overall IBS symptoms at 6 months
